# Relationship between secondary health conditions and life satisfaction in persons with spinal cord injury: study across twenty-one countries

**DOI:** 10.1007/s11136-023-03376-3

**Published:** 2023-03-02

**Authors:** Tomasz Tasiemski, Jolanta Kujawa, Piotr Tederko, Sara Rubinelli, James W. Middleton, Ashley Craig, Marcel W. M. Post

**Affiliations:** 1 Department of Adapted Physical Activity, Faculty of Sport Sciences, Poznań University of Physical Education, ul. Królowej Jadwigi 27/39, 61-871 Poznań, Poland; 2 Department of Medical Rehabilitation, Faculty of Health Sciences, Medical University of Łódź, Łódź, Poland; 3 Department of Rehabilitation, Faculty of Medicine, Medical University of Warsaw, Warsaw, Poland; 4 Department of Health Science and Medicine, University of Lucerne and Swiss Paraplegic Research, Nottwil, Switzerland; 5 John Walsh Centre for Rehabilitation Research, Kolling Institute, Royal North Shore Hospital, St Leonards, NSW Australia; 6 Faculty of Medicine and Health, University of Sydney, Sydney, NSW Australia; 7 Center of Excellence for Rehabilitation Medicine, UMC Utrecht Brain Center, University Medical Center Utrecht and De Hoogstraat, Utrecht, The Netherlands; 8 Department of Rehabilitation Medicine, University of Groningen, University Medical Center Groningen, Groningen, The Netherlands

**Keywords:** Spinal cord injury, Life satisfaction, Secondary health conditions, Treatment, Internationality

## Abstract

**Purpose:**

To determine the relationships between impact of secondary health conditions (SHCs), treatment of SHCs, and life satisfaction (LS) following spinal cord injury (SCI) across 21 countries. Hypotheses were as follows: (1) Persons with SCI and fewer SHCs report higher LS and (2) Persons who receive treatment for SHCs report higher LS than those who do not receive treatment.

**Methods:**

Cross-sectional survey, including 10,499 persons with traumatic or non-traumatic SCI aged 18 years or older and living in the community. To assess SHCs, 14 items adapted from the SCI-Secondary Conditions Scale were used (range 1–5). SHCs index was calculated as the mean of all 14 items. LS was assessed using a selection of 5 items from the World Health Organization Quality of Life Assessment. LS index was calculated as the mean of these 5 items.

**Results:**

South Korea, Germany, and Poland exhibited the highest (2.40–2.93) and Brazil, China, and Thailand the lowest (1.79–1.90) impact of SHCs. Indexes for LS and SHCs were inversely correlated (– 0.418; *p* < 0.001). Mixed Model Analysis showed that the fixed effect (key predictors of the study) of SHCs index (*p* < 0.001) and the positive interaction between SHCs index and treatment (*p* = 0.002) were significant determinants of LS.

**Conclusion:**

Persons with SCI across the world are more likely to perceive better LS if they experience fewer SHCs and receive treatment for SHCs, in comparison to those who do not. Prevention and treatment of SHCs following SCI should be a high priority in order to improve the lived experience and enhance LS.

**Supplementary Information:**

The online version contains supplementary material available at 10.1007/s11136-023-03376-3.

## Introduction

Spinal cord injury (SCI) results in disruption of the nervous system and its pathways and can have considerable physical, functional, and emotional consequences for an individual’s life. Persons with SCI are likely to experience serious health problems associated with this condition [1].

Secondary health conditions (SHCs) have been defined as “physical or psychological health conditions that are influenced directly or indirectly by the presence of a disability or underlying physical impairment” [2, p. 374]. The prevalence of SHCs in persons with SCI has been studied extensively [3–7]. Recent research has identified the most commonly reported SHCs by persons with SCI, including sexual problems, chronic pain, bladder dysfunction, spasms, joint and muscle pain, bowel dysfunction, cardiovascular problems, contractures, urinary tract infections, pressure ulcer, and postural hypotension; however, the frequency of these health conditions varied greatly among these studies [3, 4, 6, 7]. The severity of the SHCs is associated with problems, such as higher risk of mortality or psychological disorders [8].

Living with SHCs following SCI has been shown to negatively influence life satisfaction (LS), which is the personal perception of quality of life (QoL) [9]. Frequently, studies equate the two terms LS and QoL and use the concept of LS to assess QoL. Although QoL can encompass both personal and societal perceptions of what constitutes overall well-being [10], the use of LS for measuring QoL is consistent with the definition of QoL as defined by the World Health Organization Quality of Life Assessment project. That is, “an individual’s perception of his/her position in life in the context of the culture and value systems in which he/she lives and in relation to his/her goals, expectations, standards, and concerns” [11, p. 153]. Previous studies revealed that several SHCs were independently associated with lower LS or QoL, in particular neurogenic pain, pressure sores/ulcers, problematic spasticity, bowel, and bladder dysfunction [5–7, 12, 13]. Consequently, it is clear that preventive measures such as regular check-ups or patient education on self-management and early identification of SHCs are invaluable in order to more effectively avoid medical complications following SCI.

Unfortunately, treatment following SCI is not equitable nor uniformly available around the globe. Persons with SCI represent a high needs, high-cost group with significant unmet health care needs. These needs are mostly due to the costs of health services, transportation, and lack of available services [14]. This is common in low-, middle-, and high-income countries, where persons in lower-income groups are disproportionately affected. Specifically persons with SCI commonly have substantial unmet health needs when presenting for follow-up services and primary care, once initial rehabilitation has been completed [15–17]. A recent study reported that performance indicators for the health system were related to QoL, using data from the International Spinal Cord Injury (InSCI) community survey and furthermore, showed that the most important indicators for QoL were social attitudes and access to health care services [18]. Importantly, people with SCI are frequent users of health services, and this makes them highly dependent on the performance of the health system [19, 20]. The combination of unmet health needs in persons with SCI and their high dependency on the health care system for meeting these needs illustrate the importance of understanding the relationship between SHCs and available treatment for particular health problems.

The present study builds on previous InSCI studies, investigating the relationship between SHCs and LS following SCI in twenty-one InSCI countries with different access to health care services and economies [18, 21, 22]. This study focuses especially on the interaction between receiving treatment (or not) for different health problems following SCI in determining relationship between SHCs and LS, while controlling for basic demographic and clinical variables. The study tested two hypotheses: (1) Persons with SCI with fewer health problems report higher LS and vice versa and (2) Those persons with SCI who receive treatment for SHCs report higher LS than those who do not receive treatment for their health problems.

## Methods

### Design

This study was part of the cross-sectional InSCI community survey.

### Procedures

The InSCI survey was conducted in twenty-one participating countries simultaneously between January 2017 and May 2019 [23, 24]. Due to the absence of a central registry of persons with SCI in many countries, the study allowed for different types of sampling frames, ranging from convenience sampling to defined random samples based on available patient databases. The InSCI study protocol therefore allowed each country to define its sampling design process for the recruitment of participants. Different response modes, including paper and pencil, online questionnaire, telephone or personal interviews, were provided to participants (see Table 1). The study adhered with the national laws and conformed to the Declaration of Helsinki with regulatory approvals obtained from the relevant Institutional Review Boards or Ethical Committees for all countries. Each national study group was responsible for ensuring this compliance. Informed consent was sought from each participant in accordance with national regulations, either in form of written consent; completion of questionnaire (considered as implicit consent); oral consent–recorded; waiver of consent; or consent provided electronically.

**Table 1:** Correlations between SHCs index and LS index in persons with SCI living in 21 InSCI countries

No.	Country/response mode	*N*	SHCs index Mean ± SD	LS index Mean ± SD	*r*	*p* value
1	South Korea/a, b, d	810	2.93 ± 0.86*	2.80 ± 0.79	– 0.313	< 0.001
2	Germany/a, b	1490	2.42 ± 0.69	3.48 ± 0.75	– 0.512	< 0.001
3	Poland/a, b, d, e	922	2.40 ± 0.72	3.39 ± 0.67	– 0.415	< 0.001
4	Australia/a, b, e	1487	2.33 ± 0.67	3.59 ± 0.80	– 0.506	< 0.001
5	Lithuania/a, b, d	216	2.31 ± 0.74	3.53 ± 0.73	– 0.329	< 0.001
6	Italy/a, b, d	194	2.30 ± 0.64	3.27 ± 0.80	– 0.654	< 0.001
7	Japan/a	288	2.25 ± 0.69	3.15 ± 0.73	– 0.390	< 0.001
8	Morocco/a, d, e	385	2.24 ± 0.59	3.16 ± 0.73	– 0.269	< 0.001
9	Spain/a, d	390	2.21 ± 0.63	3.40 ± 0.84	– 0.446	< 0.001
10	Romania/a, b, d	206	2.14 ± 0.59	3.69 ± 0.66	– 0.347	< 0.001
11	Malaysia/a, d	278	2.13 ± 0.63	3.80 ± 0.70	– 0.283	< 0.001
12	Norway/a, b, c, e	581	2.07 ± 0.59	3.68 ± 0.66	– 0.472	< 0.001
13	USA/a, b, c, e	199	2.07 ± 0.56	3.77 ± 0.68	– 0.476	< 0.001
14	Greece/a, b, d	185	2.05 ± 0.65	3.55 ± 0.84	– 0.553	< 0.001
15	France/a, b	381	2.05 ± 0.50	3.38 ± 0.73	– 0.525	< 0.001
16	Netherlands/a, b	232	2.03 ± 0.60	3.57 ± 0.69	– 0.493	0.002
17	South Africa/a, d	199	1.98 ± 0.60	3.62 ± 0.66	– 0.215	< 0.001
18	Indonesia/b, d	194	1.97 ± 0.69	3.44 ± 0.72	– 0.223	0.002
19	Thailand/a, d	308	1.90 ± 0.66	3.57 ± 0.65	– 0.265	< 0.001
20	China/b, c, d, e	1354	1.79 ± 0.74	3.20 ± 0.62	– 0.483	< 0.001
21	Brazil/a, d, e	200	1.79 ± 0.54	3.39 ± 0.77	– 0.219	0.002
	Total (Mean)	10,499	2.23 ± 0.74	3.40 ± 0.76	– 0.418	< 0.001

Response mode*:* a—paper–pencil; b—online; c—smart phone; d—personal interview; e—telephone interview

*Ranking of InSCI countries according to SHC index in descending order

### Participants

A total of 12,591 persons with traumatic or non-traumatic SCI, aged 18 years or older, living in the community, and able to respond to one of the available language versions of the questionnaire were included in the InSCI study. Based on a power analysis, a minimal number of 200 participants per country were recruited [23]. The authors of the present study were granted access to this study sample by the InSCI Study Center at the Swiss Paraplegic Research in Nottwil, Switzerland. For the current analysis, 1530 participants from Switzerland were excluded due to the use of a different response scale for the SHCs (range 1–4) in Switzerland (in distinction to a scale range from 1 to 5 used in all other countries) and a further 562 participants were excluded because they had more than 3 missing values on the SHCs measure. This left a total number of 10,499 participants with SCI representing the following twenty-one countries: Australia, Brazil, China, France, Germany, Greece, Indonesia, Italy, Japan, Lithuania, Malaysia, Morocco, Netherlands, Norway, Poland, Romania, South Africa, South Korea, Spain, Thailand, and the USA. A prior analysis by the authors revealed some differences between those who were included (*N* = 10,499) and those excluded (*N* = 562) in relation to level of SCI (paraplegia: 61.9% vs 56.8%; *p* = 0.022; Cramer’s *V* = 0.022), years’ post-injury (mean = 12.2 years, SD = 11.4 vs mean = 15.7 years, SD = 13.7; *p* < 0.001; Cohen’s *d* = 0.308), and age (mean = 50.1 years, SD = 15.0 vs mean = 57.2 years, SD = 16.5; *p* < 0.001; Cohen’s *d* = 0.473). Whereas, there were no differences between these groups for etiology of SCI (*p* = 0.620), gender (*p* = 0.168), and severity of SCI (*p* = 0.502). However, it should be noted that comparison of such large samples are likely to show some differences. The details of these analysis have been included as online-only supplementary file (Supplementary Table).

### Measures

Participants completed a 125-item self-report questionnaire covering socio-demographic and lesion characteristics, together with questions on activities and participation, environmental and personal factors, health service utilization, and an appraisal of their health and well-being.

To assess the impact of SHCs, a selection of 14 items from the SCI-Secondary Conditions Scale (SCI-SCS) was used [25]. The SCI-SCS has been shown to demonstrate acceptable reliability and validity as a measure in the SCI population [26]. The 14 items concern: sleep problems, bowel dysfunction, urinary tract infections, bladder dysfunction, sexual dysfunction, contractures, spasticity, pressure sores/ulcers, respiratory problems, injury caused by loss of sensation, circulatory problems, autonomic dysreflexia, postural hypotension, and pain. Responses were provided on a 5-point Likert scale ranging from 1 = no problem to 5 = extreme problem. The SCI-SCS response options were modified for the InSCI questionnaire based on advice from the International Scientific Advisory Committee for the study, as it was felt that the original scoring system, incorporating concepts of both severity and frequency of a health condition (i.e., significant or chronic, moderate or occasional, mild or infrequent, and no problem), would make the results more difficult to interpret. Regarding the number of items: two items were omitted, ‘diabetes’ because that is a more remote consequence of SCI and ‘heterotopic ossification’ because of unknown reasons. The two pain items (‘chronic pain’ and ‘joint and muscle pain’) were considered conceptually overlapping items and merged into one pain item. Finally, one item ‘sleep’ was added.

All participants with more than 3 missing answers on SHCs measure were excluded from this study and the SHCs index was therefore calculated as the mean of at least 11 out of 14 items. A higher SHCs index indicates a higher rate of health problems (range 1–5). The 14 items were accompanied by an additional question asking if the participant had received treatment for the SHCs (with option of ‘yes/no’ answer). The variable ‘Treatment index’ was calculated as sum of all responses ‘yes’ to 14 specific health problems, that is, total score of all SHCs that were treated (range 0–14).

To assess LS, five items from the World Health Organization Quality of Life Assessment-5 (WHOQOL-5) were selected [27]. This was specifically developed for cross-cultural purposes and is currently available in 36 languages. The five items assess satisfaction with overall QoL, health, activities of daily living, personal relationships, and living conditions. A 5-point Likert scale ranging from 1 = very dissatisfied to 5 = very satisfied was employed. LS index was calculated as the mean of all 5 items (range 1–5). A higher LS index indicates higher LS. Research suggests that the WHOQOL-5 measure is crossculturally valid in persons with SCI [21, 27].

Age when completing the assessment, age at onset of SCI, and years’ post-injury were calculated from the questionnaire. Other factors included male or female, paraplegia or tetraplegia, complete (no motor function and sensation below the level of lesion) versus incomplete (motor and/or sensory sparing), and etiology of SCI (traumatic and non-traumatic). Income was assessed in deciles of the respective country’s income distribution. Years of education was assessed in total years of formal education before and after onset of SCI, including school and vocational training.

### Statistical analyses

Cronbach’s alpha coefficients of the LS and SHCs indexes for all InSCI countries were calculated [28]. The alpha coefficient of the LS scale was 0.81 (range across countries: 0.74–0.90) and that of the SHCs scale was 0.84 (range: 0.68–0.90). Descriptive statistics were used to present socio-demographic characteristics and the score distributions of the LS and SHCs index. Continuous variables were presented as a mean and standard deviation (SD). Categorical variables were presented in a frequency and percentage format. The coefficient of determination (R-squared) was used to assess the significance and strength of relationships between SHCs and LS. In order to assess association between the occurrence of a particular health problem and its treatment, Cramer’s *V* was used, which is an effect size measurement for the chi-square test of independence (range 0–1) [29]. The estimated effect sizes that indicate the extent of dissimilarity between the individuals who were included in the analysis and those excluded due to missing data were reported as Cramer’s *V* (for categorical variables) or Cohen’s *d* (for continuous variables).

Mixed Model Analysis (Hierarchical Linear Models) were used to test the study hypotheses. The dependent variable was the LS index. Predictors in the analysis were categorized into random effect and fixed effects. Random effects refer to variables that are not the main focus of a study but may impact the dependent variable, and fixed effects are key predictors of the study. The random effect (country) was introduced to account for the relative correlation between data points that fall within one hierarchical level, whereby participants with SCI from one country are more alike than participants with SCI across 21 countries. The fixed effects contained the SHCs index, received treatment, and their interaction, sex, age, level, and extent (completeness) of SCI, years’ post-injury, and etiology of SCI. All statistical analyses were performed using the IBM SPSS Statistics.^1^

## Results

Altogether 12,591 persons with SCI participated in the InSCI study and data from 10,499 individuals with SCI were analyzed. The mean age for all of the study participants at the time of the questionnaire was 50 years (SD = 15.0), 73% were male, and mean time since onset of injury or disease was 12 years (SD = 11.4). The respondents had complete paraplegia (28%), incomplete paraplegia (34%), complete tetraplegia (10%), or incomplete tetraplegia (28%). Half (52%) of respondents were married, they had on average 12 years (SD = 5.3) of education, and 58% had a monthly income below the fifth decile of country’s income distribution.

The mean values for SHCs and LS indexes are presented in Table 1. The highest rate of health problems was identified in South Korea, Germany, and Poland (range: 2.40–2.93). In contrast, the lowest rate of health problems was noted in Brazil, China, and Thailand (range: 1.79–1.90). With respect to LS, the highest level of LS was shown in Malaysia, the USA, and Norway (range: 3.68–3.80) and the lowest in South Korea, Japan, and Morocco (range: 2.80–3.16).

Significant negative correlations were found between the LS index and SHCs index for all InSCI countries and in the whole study sample (*r* = – 0.418; *p* < 0.001; with *r*^2^ explaining 17.5% of the variability). Participants with fewer health problems reported higher LS. This finding confirmed Hypothesis 1.

The percentage of people with SCI who received treatment for a particular SHC is shown in Table 2. The more serious the perceived health problem, the higher the percentage of persons receiving treatment for that problem, with exception of sexual dysfunction. Also, for each health problem, a small percentage of those who reported having no health problem reported receiving treatment (see Table 2). Data revealed that not all people with SCI who experienced SHCs rated as an extreme problem received treatment. This percentage treated was highest for urinary tract infections (84.1%) followed by pressure sores/ulcers (81.0%) and lowest for sexual dysfunction (14.0%). The strongest correlation was identified between treatment and occurrence of pressure sores/ulcers (Cramer’s *V* = 0.69) and urinary tract infections (Cramer’s *V* = 0.68), while weakest with occurrence of sexual dysfunction (Cramer’s *V* = 0.19).

**Table 2:** Percentage of people with SCI who received treatment for separate SHCs in 21 InSCI countries

Health problems	People with SCI who received treatment (%)					Cramer’s *V*	*p* value
	1 no problem	2	3	4	5 extreme problem		
Sleep problem	2.8	10.2	22.7	33.6	46.5	0.38	< 0.001
Bowel dysfunction	4.5	18.7	35.7	49.4	57.9	0.43	< 0.001
Urinary tract infection	4.5	44.5	67.0	79.7	84.1	0.68	< 0.001
Bladder dysfunction	4.2	28.5	45.6	57.6	64.6	0.51	< 0.001
Sexual dysfunction	1.7	12.9	16.2	15.8	14.0	0.19	< 0.001
Contractures	2.3	18.4	30.7	43.0	48.7	0.41	< 0.001
Spasticity	3.0	19.8	37.8	49.7	61.2	0.45	< 0.001
Pressure sores/ulcers	2.4	34.9	56.9	71.2	81.0	0.69	< 0.001
Respiratory problems	2.4	18.5	35.9	47.7	64.0	0.50	< 0.001
Injury/loss of sensation	1.5	19.5	34.5	48.5	52.6	0.53	< 0.001
Circulatory problems	2.5	16.2	28.4	39.2	53.5	0.43	< 0.001
Autonomic dysreflexia	1.4	14.4	26.8	38.1	48.8	0.45	< 0.001
Postural hypotension	1.3	10.3	18.9	34.8	43.3	0.40	< 0.001
Pain	2.9	19.7	38.2	52.0	66.2	0.48	< 0.001

The type III tests of fixed effects showed that the SHCs index (*F*(9966) = 1852.1, *p* < 0.001), as well as the interaction between the SHCs index and treatment (*F*(9961) = 11.3, *p* < 0.001) were significant determinants of LS. This significant interaction coefficient means that persons with SCI who experience a higher burden of SHCs have higher perceived LS if they report having received treatments for these health problems, independent of severity of SHCs. This finding confirmed Hypothesis 2. The model also included fixed effects of etiology and level of SCI and years post-injury. Participants with traumatic injury, *F*(9966) = 20.8, *p* < 0.001, paraplegia, *F*(9966) = 45.5, *p* < 0.001, and with more years since SCI, *F*(9966) = 102.3, *p* < 0.001, all had higher LS than those with non-traumatic injury, tetraplegia, and less years since SCI, respectively. The following fixed effects were not significant: treatment when considered independently (*p* = 0.658), extent of SCI (*p* = 0.144), age (*p* = 0.091), and sex (*p* = 0.836). The results of Mixed Model Analysis (i.e., estimates of fixed effects and covariance parameters) are summarized in Table 3. The random effect of country was also significant (*Z* = 3.13, *p* = 0.002), showing that its inclusion was warranted.

**Table 3:** Fixed and random effects for mixed model analysis with LS index as dependent variable

Model term	Fixed effects*			
	Estimate	Std. error	T test	*p* value
Intercept	3.282	0.049	67.23	< 0.001
SHCs index	– 0.357	0.008	– 43.04	< 0.001
Treatment	0.004	0.008	0.44	0.658
SHCs x Treatment	0.019	0.006	3.37	< 0.001
Traumatic	0.082	0.018	4.56	< 0.001
Paraplegia	0.094	0.014	6.74	< 0.001
Complete	0.021	0.015	1.46	0.144
Age	0.012	0.007	1.69	0.091
Years post-injury	0.077	0.008	10.12	< 0.001
Female	0.003	0.015	0.21	0.836

	Random effect*			
	Estimate	Std. error	Wald *Z*	*p* value
Residual	0.416	0.006	70.55	< 0.001
Country	0.041	0.013	3.13	0.002

*Dependent variable: LS index

## Discussion

A comparison between the included and excluded participants revealed that the excluded participants were on average older and had a shorter time since onset of SCI. However, the level of missing data was a small percentage of the overall sample and therefore is not expected that missing data have influenced the estimated coefficients. This study investigated the relationship between SHCs and LS in persons with SCI living in twenty-one countries representing all six WHO regions with diverse access to health care services and economies. The results of this study show that experiencing a greater burden of SHCs is associated with decreased LS, confirming our first hypothesis. The second hypothesis of the present study that persons with SCI receiving treatment for SHCs report higher LS than those who do not receive treatment for health problems was also proven.

The results of this study are consistent with previous studies reporting higher LS in persons with SCI experiencing fewer SHCs [5–7, 12, 13]. Also, the most commonly reported health problems following SCI in our study, such as urinary tract infections, pressure sores/ulcers, and pain, are similar to those previously reported [3, 4, 6, 7]. Building on previous InSCI studies [18, 21], the main added value of the present analysis is the inclusion of data on whether treatment was received or not for particular health problems contributes significantly to the relationship between SHCs and LS, while controlling for country as random effect.

The positive association between number of SHCs treated and LS highlights the importance of access to appropriate health care for persons with SCI. However, it is quite troubling that many persons with SCI who experience serious health problems cannot rely on the available country health care system. The collected data revealed that less than 50% of persons with SCI who report extremely severe problems with sleep, contractures, autonomic dysreflexia, postural hypotension, and sexual dysfunction received treatment. Our results extend previous research showing that relative frequencies of treatment of SHCs following SCI were low (median 44%, interquartile range 25–64%), even for significant or chronic problems [4]. Notably, our study found that only 14% of persons with SCI and extreme sexual dysfunction were treated, flagging a serious unmet need. Regaining sexual function has previously been reported as the highest priority for persons with paraplegia in regard to enhancing QoL and as the second priority for persons with tetraplegia, just behind recovery of arm and hand function [30]. This clearly shows that health care systems around the world often fail to adequately address the rehabilitation needs voiced by persons with SCI. This is in line with a report on health care needs in twenty InSCI countries in which health care cost, transportation, and service availability were identified as the most important unmet needs, and persons with SCI in lower-income groups were disproportionately impacted by this situation [14].

Another InSCI study has found that persons with SCI reporting unmet health care needs, being a smoker, being a female, having a complete lesion, and a traumatic injury exhibited significant associations with comorbidity [22]. Previous outcomes from InSCI project also showed that persons with SCI in higher-income groups have lived more years with the injury and experienced fewer comorbidities than people in poorer-income groups [31]. All of the above findings indicate that the living situation of people with SCI is strongly influenced by the performance of the health system [18]. Our findings in persons with SCI can serve as a case model for other chronic diseases, highlighting how such a serious and challenging health condition does not receive enough attention at the public health system level around the world.

### Study strengths and limitations

The InSCI study is the first worldwide SCI survey conducted across 21 countries with varying economic status. InSCI also provides data demonstrating a relationship between SHCs and LS in persons with SCI from these 21 countries. A further strength was the employment of a valid LS measurement tool with cross-cultural sensitivity. Furthermore, appropriate standards were determined for valid translation of the questionnaire in the respective languages. These processes strengthened the validity of comparisons across the 21 countries.

However, the data in the questionnaire on health conditions were self-reported and it was not possible to verify by health professionals, while willingness to disclose sensitive health data may be related to cultural issues, and this may have introduced reporting bias. Another limitation was the variation in sampling frames and use of convenience samples in many of the participating countries, and this may have influenced comparisons between the countries and therefore, the generalizability of the results. Also, given the lack of population-based data on persons with SCI around the world, we were not able to estimate the representativeness of the InSCI sample on a country-by-country basis. Additionally, a wide variation in sample sizes across countries existed, and countries with larger samples were weighted more in the total scores. The different response modes, from online, paper–pencil questionnaires to telephone, or personal interviews could well have influenced the quality of data, such as LS. We have no evidence regarding the equivalence of different response modes using the InSCI survey.

### Practical implications

Due to high prevalence of SHCs and their relation to LS following SCI, development of strategies for improved health promotion, supported self-management, surveillance, and early intervention are of great importance. Our findings support the outcomes of previous research where preventive measures of primary care following SCI include regular follow-up by specialized teams and annual comprehensive health examination, as well as access to disability-specific expertise in the form of specialists, regarding common SHCs, such as pain and bowel and bladder complications [15]. Apart from that, there are at least several other options for increasing knowledge about own health status among persons with SCI, such as multimedia patient education resources (labeled SCI-U), developed by rehabilitation professionals and consumers from Canada available in the form of ‘SCI and you’ courses [32]. Alternatively, resources provided by the International Spinal Cord Society include consumer modules (elearnSCI) available online upon earlier registration with this platform.^2^ An example of a program tackling prevention of SHCs following SCI is also the community-based Active Rehabilitation program—a grassroots transfer of practical life and social skills from experienced and active individuals with SCI (peer mentors) to newly-injured individuals [33, 34]. Education sessions during Active Rehabilitation camps are intended to help participants acquire or update knowledge that would allow them to optimally manage their health. These sessions target prevention of pressure sores/ulcers, prevention of urinary tract infections, bowel management, and sexuality and fertility disorders. This peer-based program has been introduced in more than twenty countries in Europe, Africa, and Asia [34–37].

## Conclusion

The findings of the present study underline the need for community engagement in health care following SCI, so that people’s health needs will be identified and addressed early. This requires enabling an infrastructure for dialogue, based on the gold standards of shared decision-making and empowerment through education of people with SCI, improving health literacy to be able to express their needs and moreover to navigate a complex health care system to address their needs. Clearly, there are countries where tailored health professional–patient interaction is still not routine. Thus, it is not surprising that health needs may remain unmet and that people with SCI experience a high burden of SHCs, not resolved by appropriate treatments. Prevention of SHCs following SCI should be a high priority to improve the lived experience and enhance LS, while reducing morbidity, rehospitalization, and health care costs.

## Supplementary Information

Below is the link to the electronic supplementary material.

Supplementary file1 (DOCX 15 KB)

Supplementary file2 (PDF 270 KB)

## Data Availability

Owing to our commitment to the study participants and their privacy, datasets generated during the current study are not made publicly available but can be provided by the Study Center based on reasonable request (insci@paraplegie.ch).

## Footnotes

1 IBM SPSS Statistics 28.0; IBM Corp., Armonk, New York, USA.

2 https://www.elearnsci.org/.
