# Supplementary material for: Relationship between secondary health conditions and life satisfaction in persons with spinal cord injury: study across twenty-one countries
Source: Qual Life Res. 2023 Mar 2;32(7):2069–77. doi: 10.1007/s11136-023-03376-3 (PMC10241701; doi:10.1007/s11136-023-03376-3)
Supplement: Supplementary file 1 — Supplementary file1 (DOCX 15 KB) [file 11136_2023_3376_MOESM1_ESM.docx]

**Supplementary Table** Comparison between InSCI study participants included and not included in the analysis

|  | **Study participants** | |  |  |  |
| --- | --- | --- | --- | --- | --- |
| **Demographic and injury characteristics** | **Included (N=10,499)** | **Not included (N=562)** | **Test**  **result^1)^** | ***P* value** | **Effect size^2)^** |
| *Gender*  Male  Female | 95.3%  94.6% | 4.7%  5.4% | *x^2^*=1.903 | 0.168 | *V*=0.013 |
| *Etiology*  Traumatic  Non-traumatic | 95.2%  95.4% | 4.8%  4.6% | *x^2^*=0.245 | 0.620 | *V*=0.005 |
| *Level of SCI*  Paraplegia  Tetraplegia | 95.7%  94.8% | 4.3%  5.2% | *x^2^*=5.263 | 0.022 | *V*=0.022 |
| *Severity of SCI*  Complete  Incomplete | 95.2%  95.4% | 4.8%  4.6% | *x^2^*=0.451 | 0.502 | *V*=0.006 |
| *Age* Mean ± SD | 50.1 ± 15.0 | 57.2 ± 16.5 | *t*=10.754 | <0.001 | d=0.473 |
| *Years post-injury* Mean ± SD | 12.2 ± 11.4 | 15.7 ± 13.7 | *t*=6.687 | <0.001 | d=0.308 |

***Note***

^1)^ T-test (*t*); Chi square test (*x^2^*)

^1)^ Cramer’s *V*; Cohen’s d
