# Supplementary material for: Relationship between secondary health conditions and life satisfaction in persons with spinal cord injury: study across twenty-one countries
Source: Qual Life Res. 2023 Mar 2;32(7):2069–77. doi: 10.1007/s11136-023-03376-3 (PMC10241701; doi:10.1007/s11136-023-03376-3)
Supplement: Supplementary file 2 — Supplementary file2 (PDF 270 KB) [file 11136_2023_3376_MOESM2_ESM.pdf]

International Spinal Cord Injury (InSCI) Community Survey 2018
Ethics committees or review boards approvals in the 22 InSCI countries

Country	Name of ethics committee or institutional board responsible for ethics approval of the InSCI study	Approval number	Approval date	Form of consent
Australia	Northern Sydney Local Health District Human Research Ethics Committee;<br>Australian Institute of Health and Welfare Ethics Committee	HREC/16/HAWKE/495;<br>EO2017/1/341	June 7 2017;<br>Jan 4 2018	D
Brazil	Comissão de Ética para Análise de Projetos de Pesquisa do Hospital das Clínicas da Faculdade de Medicina da Universidade de São Paulo, CAPPesq	97049118.8.0000.0068	Nov 8 2018	A
China	Ethics committees of the first affiliated hospital of Nanjing Medical University, Nanjing, Jiangsu Province;<br>Ethics Committee of Sichuan University, Sichuan Province, Chengdu	No. 2018-SR-004 (Jiangsu)<br>No. K2017053 (Sichuan)	March 7 2018;<br>Jan 22 2018	B, C
France	Comité de Protection des Personnes	Ref : 180304	April 10 2018	A
Germany	Ethic Committee of Hannover Medical School	7374	Feb 13 2017	B
Greece	Scientific/Ethical Committee of General Hospital 'G. Gennimatas' Athens	20257/1.8.2016	Aug 1 2016	A, B
Indonesia	Health Research Ethics Committee, National Institute of Health Research and Development	LB.02.01/2/KE.342/2017	15 Nov 2017	A
Italy	Comitato Etico Interaziendale AOU 'Maggiore della Carità' di Novara, ASL BI, ASL NO, ASL VCO	ItaSCI, 1, 25-01-2018	April 27 2018	A
Japan	Research Ethics Committee of Wakayama Medical University	2079	July 12 2017	A
Lithuania	Vilnius Regional Committee for the Ethics of Biomedical Research	158200-17-907-421	Mai 9 2017	A, B
Malaysia	Medical Research and Ethics Committee, Ministry of Health	NMRR-16-2747-28885(IIR)	July 14 2017	A
Morocco	Hospital and University Ethics Committee of Fez	03/17	July 20 2017	A
Netherlands	Medical Ethics Board University Medical Center Utrecht	WAG/mb/17/024763	Aug 16 2017	A
Norway	Regional Committee for Medical and Health Research Ethics, South East	2016/1184/REK sør-øst	Sept 21 2016	A, B
Poland	Bioethical Committee of the Medical University of Lodz	RNN/198/16/KE	July 12 2016	A, B
Romania	Ethical Committee of Rehabilitation Hospital Felix Spa	2228/06.03.2017	March 3 2017	A, B
South Korea	Institutional Review Board of National Rehabilitation Center	NRC-2016-05-039	Nov 9 2016	A, B
South Africa	Biomedical Science Research Ethics Committee of the University of the Western Cape	BM/16/3/24	Oct 24 2016	A, C (recorded)
Spain	Ethical Committee of Hospital Universitari Vall d'Hebron, Hospital Universitario de Cruces, Hospital Universitario Materno Infantile de Gran Canaria, Hospital Universitario Virgen del Rocio	PR(ATR)285/2016	Oct 2016	A
Switzerland	Ethical Committee of Northern and Central Switzerland	11042 PB_2016-02608	Dec 21 2016	A, B
Thailand	Ethical Committee of Faculty Medicine, Chiang Mai University	REH-2559-04167	Dec 9 2016	A
United States	University of Vermont Institutional Review Board	CHRBSS:16-574	Nov 21 2017	A, E

Note: A=written consent; B=questionnaire completion considered as implicit consent; C=oral consent; D=waiver of consent; E=electronic consent
